# Variation of Corrosion Rate, V_corr_, during the Carbonation-Induced Corrosion Propagation Period in Reinforced Concrete Elements

**DOI:** 10.3390/ma17010101

**Published:** 2023-12-24

**Authors:** Javier Sánchez Montero, Pascual Saura Gómez, Julio Emilio Torres Martín, Servando Chinchón-Payá, Nuria Rebolledo Ramos

**Affiliations:** 1 Instituto Eduardo Torroja de Ciencias de la Construcción (IETcc-CSIC), Calle de Serrano Galvache, 4, 28033 Madrid, Spain; javier.sanchez@csic.es (J.S.M.); juliotorres@ietcc.csic.es (J.E.T.M.); servando@ietcc.csic.es (S.C.-P.); nuriare@ietcc.csic.es (N.R.R.); 2 Departamento de Construcciones Arquitectónicas, Universidad de Alicante, 03690 San Vicente del Raspeig, Alicante, Spain

**Keywords:** corrosion, carbonation, serviceability limit state, ultimate limit state, service life

## Abstract

The structural systems of residential buildings in many developed countries have widely utilized reinforced concrete as the most common solution in construction systems since the early 20th century. The durability of reinforced concrete columns and beams is compromised, in most cases, by pathologies caused by the corrosion of their reinforcements. This study analyses the corrosion processes induced by carbonation in 25 buildings with reinforced concrete structures. The models estimate the service life of reinforced concrete elements by differentiating between the initiation period and the propagation period of damage, considering two possible stages: the time of corrosion propagation until the cracking of the concrete cover, and the time of propagation until a loss of section is considered unacceptable for structural safety. However, the mathematical expressions that model the propagation periods consider the same corrosion rate in both cases. This research has found that the average corrosion rate in elements with an unacceptable loss of reinforcement section was in the order of 8 times higher than the corrosion rate in cracked columns and beams without a loss of reinforcement. This opens up a path to improve the definition of the different stages experienced by a reinforced concrete element suffering corrosion of its reinforcements due to carbonation, because once the concrete has cracked, the corrosion rate increases significantly.

## 1. Introduction

### 1.1. Background

Reinforced steel in reinforced concrete is protected by a physical layer, which is the concrete cover, and a chemical layer, which is the alkaline protection with an approximate pH of 13, present in the concrete pores provided by sodium and potassium oxides and hydroxides generated during cement hydration. If the alkaline layer is neutralized, corrosion can occur. This basic idea about corrosion has been extensively studied in specialized treatises such as Tuutti [1] and Broomfield [2] in concrete, and Fontana and Greene in engineering [3], and in textbooks like Atkins and De Paula [4].

When studying corrosion processes caused solely by the carbonation of the cover, without the presence of chlorides, there are many factors that influence the movement of aggressive agents from the concrete surface to the reinforcement and the evolution of oxidation reactions once the steel has lost its protective layer [5,6,7,8,9,10]. According to the Tuutti model [1], these phenomena define the durability of reinforced concrete and have been classified by the European Standard Eurocode 2 [11] and Spanish Structural Code [12] into two periods or stages: the initiation of corrosion, in which the reinforcement is passivated, but chlorides and carbonation are transported through the concrete cover, and the propagation of corrosion, which begins when the steel is exposed to oxidation reactions.

The corrosion of reinforcements is one of the pathologies that will affect the service life [13,14,15,16,17,18] of reinforced concrete structures. It is possible for oxidation reactions to continue in intensity and propagation time, generating more oxidation products that can cause further cracking and a significant loss of the initial steel section, which may become unacceptable.

### 1.2. Carbonation-Induced Corrosion

The main cause of a generalized attack on a reinforced concrete element is the penetration of CO_2_ [9,13,18,19,20,21,22,23,24,25,26,27,28,29,30,31], present in the atmosphere, through the pores of the cover, leading to a decrease in pH to values close to 9 where the passive film is no longer stable. To calculate the carbonation depth, x (mm), we use the mathematical formula x = $K_{{\mathrm{CO}}_{2}}\;\sqrt{t}$, where Kco_2_ (mm/$\sqrt{year}$) represents the carbonation coefficient that measures the penetration rate of the aggressor under specific environmental conditions and for concrete with specific characteristics [32,33,34]. Several factors, such as moisture [32,33], CO_2_ concentration [34,35], temperature, concrete quality, water/cement ratio [34], characteristic strength, porosity [13], and cement type [28], influence the carbonation process (as developed in Supplementary Materials). Consequently, more compact and stronger concrete offers greater resistance to carbonation.

#### 1.2.1. Initiation Period of Corrosion by Carbonation

The initiation period of corrosion by carbonation is defined as the time that elapses from the commissioning of the element until the corrosion damage to the reinforcement begins, and the model defined by the standard [11,12] is as follows:

(1) $$t_{init}={\left(\frac{c}{K_{{CO}_{2}}}\right)}^{2}$$

where c (x = c) is the concrete cover in mm, Kco_2_ is the carbonation coefficient in mm/√year, and it measures the penetration rate of carbonation in reinforced concrete.

#### 1.2.2. Propagation Period of Corrosion by Carbonation

The propagation period of corrosion by carbonation is considered from the onset of corrosion until an unacceptable damage occurs. In order to understand the influence of corrosion, its rate is considered negligible if it is less than 0.1 µA/cm^2^ (<1.17 µm/year); moderate if it ranges from 0.1 to 0.5 µA/cm^2^ (1.17 to 5.85 µm/year); high if it falls between 0.5 and 1 µA/cm^2^ (5.85 to 11.7 µm/year); and very high if it exceeds 1 µA/cm^2^ (>11.7 µm/year) [36,37]. The Structural Code [12] considers a corrosion rate, V_corr_, between 1 and 5 µm/year for different environments of exposure class. These carbonation-induced corrosion rates are categorized as moderate corrosion risk. In the propagation period there are two considerations:

The propagation time for cover cracking. The compounds formed because of corrosion occupy a much larger volume than the initial steel, which leads to cracks and fissures parallel to the direction of the steel bars. The time elapsed from the onset of corrosion to the cracking of the cover, as defined by the standard, is calculated as follows [11,12]:

(2) $$t_{crack,corr}=\frac{P_{corr}}{V_{corr}}=\frac{80.c}{\Phi .V_{corr}}$$

where

t_crack,corr_—time from the onset of corrosion to cover cracking, in years;
P_corr_—limiting corrosion penetration, in μm;
c—thickness of the concrete cover, expressed in mm;
Φ—diameter of the reinforcement, expressed in mm;
V_corr_—corrosion rate, expressed in μm/year.

The propagation time of corrosion for an unacceptable loss in diameter of the reinforcement. The corrosion processes continue to propagate, causing a reduction in the cross-sectional area of the reinforcement that advances towards the interior of the steel bar, progressively affecting its load-bearing capacity and posing a risk to the structural safety of the element. The time elapsed from the initiation of corrosion until the occurrence of an unacceptable reduction in the cross-sectional area of the reinforcement, as defined by a thickness ΔΦ, can be calculated according to [11,12]:

(3) $$t_{sect,corr}=\frac{\Delta \Phi }{V_{corr}}$$

where

t_sect,corr_—the time elapsed from the onset of corrosion until the occurrence of an unacceptable loss in diameter in the reinforcement, in years;
ΔΦ—the unacceptable variation in diameter of the reinforcement, expressed in μm.

### 1.3. Structural Limit States (Supplementary Materials)

The limit states are defined as situations in which the structure does not fulfil some of the functions for which it has been designed. In the nominal service life, the building and its structure can respond to the normal conditions of use of the building or infrastructure with an adequate response to the effect of actions. We can consider that this period corresponds, in terms of possible reinforcement corrosion, to the initiation period of corrosion. The serviceability limit state affects the functionality and use of the structure, the aesthetic aspects of the construction elements, and the comfort of the users. Therefore, in this state, it is necessary to consider the cracking of the reinforced concrete coverings in pillars, beams, and slabs caused by reinforcement corrosion. Based on these considerations, the age of the building in its service life until the serviceability limit state is the sum of the initiation period of corrosion and the time for corrosion propagation until the cracking of the coverings occurs. The ultimate limit state affects the stability of the structure and therefore the safety of the building’s users. The structure reaches an ultimate limit state when the corrosion of the reinforcement has caused a section loss that is deemed unacceptable to ensure structural stability. Therefore, the age of the building in its service life until the ultimate limit state is the sum of the corrosion initiation period and the propagation time of corrosion until the deemed unacceptable section loss occurs.

### 1.4. Cracking

**Figure 1:**
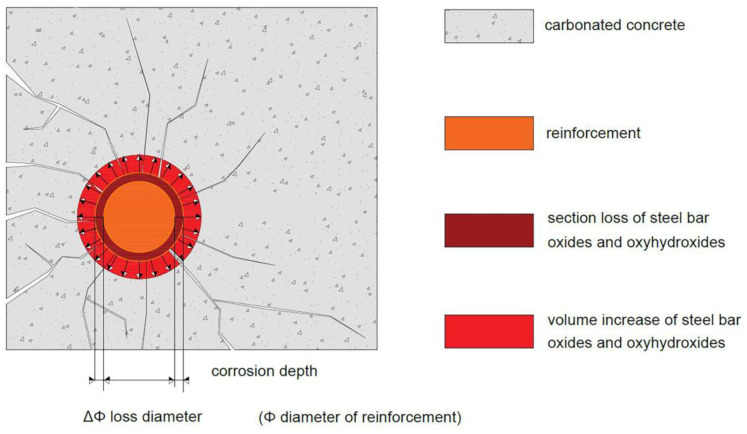
Schematic representation of corner reinforcement with corrosion (appearance of oxides and oxyhydroxides), cracking (volume increase), and loss of section.

The cracking models depend on the type of concrete fracture. Over the past decades, research has been conducted on the behaviour of rust as a cause of concrete cracking, as schematically represented in a section of the corner of an element, shown in Figure 1. Two trends or models have been established in the interpretation of this pathology: discrete cracks considered as a linear elastic behaviour [38], and diffuse cracks with a unique behaviour [39,40,41]. Other models consider finite elements with diffuse cracking and a fluid behaviour for rust [42], which is used as a basis for rust modelling. On the other hand, models with discrete cracking using embedded adaptable crack elements have also been employed [43].

In most cases, cracks do not appear until the advanced stages of corrosion due to the assumed bond between steel and concrete. The majority of oxidation products occupy a volume between two and six times the initial volume of the steel [44,45], depending on the type of generated oxide: FeO, magnetite Fe_3_O_4_, maghemite γ-Fe_2_O_3_, and hematite α-Fe_2_O_3_ can occupy approximately twice the volume of the initial Fe; lepidocrocite γ-FeOOH and iron hydroxides Fe(OH)_2_ and Fe(OH)_3_, four times the initial volume; and hydrated iron hydroxide Fe(OH)_3_ + 3H_2_O, six times the initial volume [46,47]. However, the larger volume of oxidation products can diffuse or flow through the network of pores, capillaries, and potential microcracks in the concrete, resulting in a smaller effective expansion on the concrete cover than what the initial steel actually experiences [48,49,50,51,52].

On the other hand, the stiffness of the oxide layer will depend on its composition, which increases uncertainties regarding the cracking effects that will occur. This research is based on experiences with reinforced concrete elements on which it has been verified that carbonation reached the longitudinal reinforcement (hence, the transverse reinforcement or stirrups were in a fully carbonated area), and the cracking of the cover had already been detected, with or without a loss of reinforcement section. The studied elements that did not exhibit cracking did not have carbonation throughout the entire cover of the concrete either.

### 1.5. Objectives

One of the main objectives of this study is to improve the prediction models of reinforcement corrosion induced by carbonation and to develop a methodology that supports the information and forecasts of degradation processes in reinforced concrete structures that affect their durability. The aim is to diagnose the pathologies, characteristics, and properties analysed in the condition of the structure, creating interpretation protocols for determining the current level of deterioration of the analysed element, in order to forecast its durability based on its service state and facilitate decision making in potential intervention projects. More specific objectives will involve investigating variables such as the carbonation coefficient and corrosion rate, and defining the initiation and propagation periods of corrosion. A comparison will be made between the results obtained from the models defined in the standards and those derived from real data on the condition of the structure (age, depth of carbonation, and corrosion rate). Furthermore, the Standard [12] establishes a single V_corr_ model in each exposure environment, but this work shows the important difference between the period of corrosion propagation until the cracking of the concrete cover and the period of propagation until an unacceptable loss of cross-sectional area of the reinforcement.

A debate arises regarding the assimilation of the serviceability limit state of the structure with the cracking of the concrete cover, and the ultimate limit state with the loss of cross-sectional area of the reinforcement. This study analyses all the issues that affect the interaction of concrete and steel, and the considerations that architects or engineers must assume when designing and/or calculating the affected structure, as well as the intervention criteria (Supplementary Material).

## 2. Experimental Phase

### 2.1. Buildings and Analysed Elements

**Figure 2:**
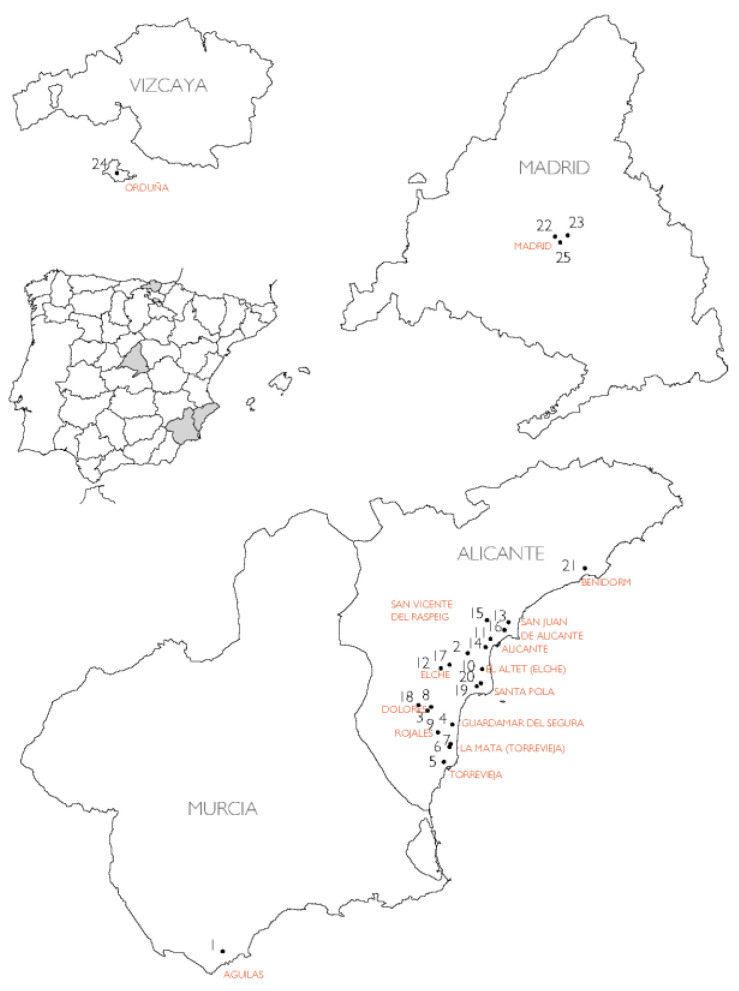
Geographic identification of the 25 buildings studied.

This investigation is conducted based on the data collected from the analysis of 143 reinforced concrete elements within 25 distinct buildings situated in Spain. Most of these buildings are in the province of Alicante, with three in Madrid, one in Murcia, and one in Vizcaya. The precise geographical distribution of these buildings can be observed in the map presented in Figure 2, and their respective identifications can be found in the Supplementary Material provided.

All considered variables are presented in a summary table (Supplementary Material) that gathers all available information and has served as the basis for conducting this research.

### 2.2. Building Age

The building’s construction date and the timing of the studies and tests conducted on each of its elements are available, thus providing information about its age. The construction date has been obtained from project data, where available, or from public information provided by the Real Estate Cadastre. Buildings with ages ranging from 9 years, in the case of a precast reinforced concrete structure where carbonation had not reached the reinforcement, to one of the earliest reinforced concrete experiences in Spain from the year 1904, with an age of 118 years for the analysed elements in 2022, have been studied. In the latter case, advanced carbonation had caused significant cracking and diameter loss in the reinforcement.

### 2.3. The Cross-Section of the Reinforced Concrete Element

a. Diameter of the reinforcement (Φ in mm). The diameter of the reinforcement was measured in situ using a calliper at the location where the reinforcement was detected using a cover meter, by creating an excavation or extracting a core sample. In all collected data, only the longitudinal reinforcements have been considered.
b. Cover (c in mm). In all cases where there was no cracking, or where cracking existed but the reinforcement had not lost a section (or the loss was negligible), columns, beams, or joists were studied. Due to the construction conditions and possible variations in cover within the same element, the nominal project cover was considered, along with the thickness of the transverse reinforcement (stirrup). In cases where the diameter of the reinforcement had decreased, the cover was measured at the corresponding point.

### 2.4. Concrete Tests

**Figure 3:**
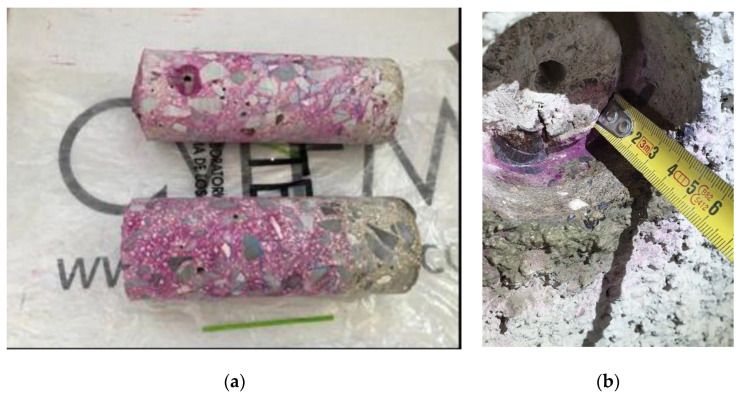
Carbonation depth testing using a phenolphthalein solution: (**a**) on a core sample; (**b**) on a drilled hole element.

**Figure 4:**
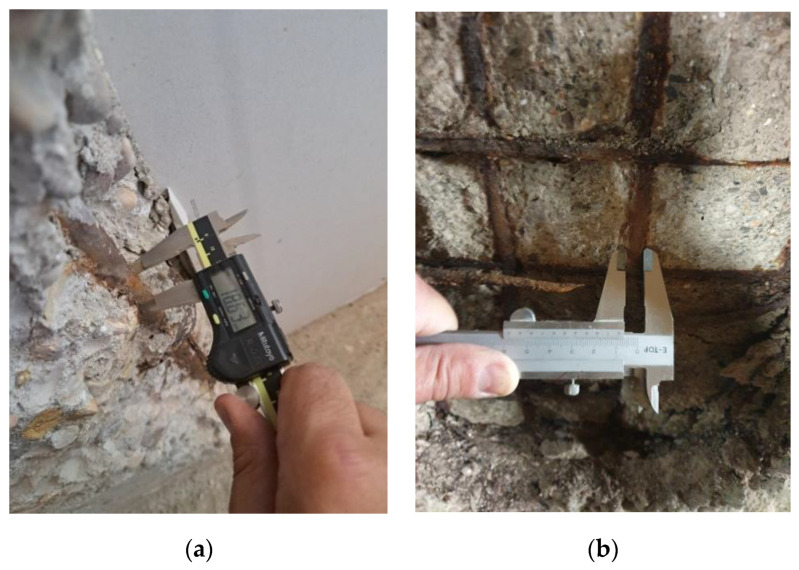
Measurement using a digital (**a**) or manual (**b**) calliper of the reinforcement diameter with cross-sectional loss.

**Figure 5:**
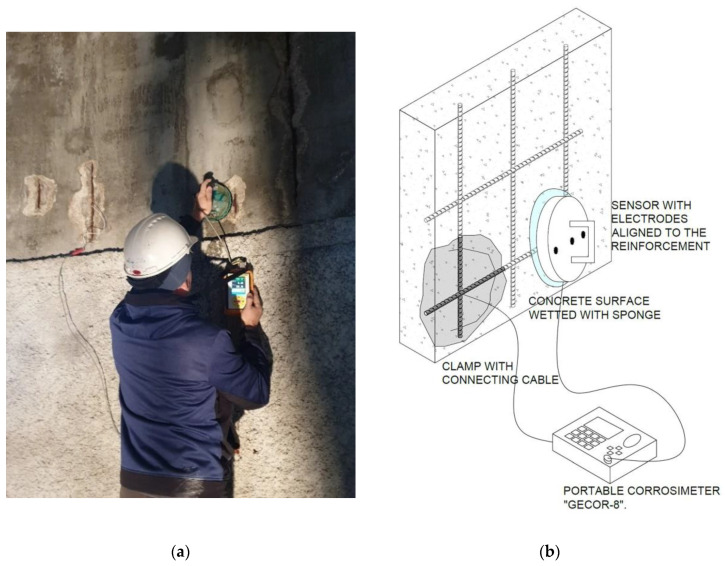
In situ corrosion rate measurement (building 24) using portable tester (**a**). Connection diagram with corrosimeter Gecor8 (**b**).

a. Characteristic compressive strength (f_cK_ in N/mm^2^). A certified laboratory carried out the determination of the compressive strength of the concrete in each case. A core sample was extracted using a rotary drilling method with a diameter of 75 mm, following standards UNE-EN 12504-1 and UNE-EN 12390-3 [53,54]. Additionally, tests were conducted on the ultrasonic propagation velocity in elements of the same building to estimate results in other homogeneous concrete elements within the building under similar conditions.
b. Carbonation depth (x in mm). In all analysed elements, the carbonation depth was measured, from the outer surface to the depth of carbonated concrete according to standard UNE-EN 14630-2007 [55]. Whether on the concrete core sample used for compressive strength testing (Figure 3a) or on the drilled hole in the same element (drill with a diameter larger than 2 cm or hole resulting from an extraction (Figure 3b), the concrete was sprayed with a solution of phenolphthalein in ethyl alcohol at a concentration of 1%. The depth was measured using a tape measure until the colour changed (pinkish tone in the non-carbonated zone and colourless concrete in the carbonated zone).
c. Cracking (0, 1, 2). In all components, the laboratory and the technician supervising the works determined the presence or absence of cracks within each component. For each instance, an in situ inspection of the reinforcement was conducted, assessing any potential reduction in the original diameter of the longitudinal reinforcements, examining their condition, and verifying the presence of corrugation and/or oxidation to ascertain any loss of cross-sectional area. Subsequently, measurements were performed accordingly. According to the data, three potential scenarios have been identified: 0: No cracking observed; 1: Cracking without significant loss of reinforcement cross-section (negligible); and 2: Cracking with measurable loss of reinforcement cross-section. According to studies and calculations made based on the Eurocodes, the serviceability limit state in a reinforced concrete structure with corrosion due to carbonation is a loss of 50 μm in reinforcement diameter [56]. Therefore, the present study analyses case 1 and case 2 with the above-mentioned threshold to differentiate whether the loss of a reinforcement cross-section is negligible or not, respectively.
d. Loss of cross-section (ΔΦ in μm). The final diameter of the reinforcements that had experienced a significant reduction in their nominal diameter was measured, indicating an unacceptable loss that poses a risk to the structural safety of the component. The measurements were taken in millimetres using a digital or mechanical calliper (and converted to micrometres in calculations), as shown in Figure 4a,b.
e. In situ corrosion rate (V_corr_ in μm/year). In cases where a significant diameter loss was detected, posing a risk to the stability and safety of the structure, the corrosion rate was measured using a portable corrosion tester (Figure 5a). The measuring equipment requires a connection circuit with one copper clamp in contact with the corroding reinforcement, a sensor with three electrodes in the same direction as the reinforcement bar, and a water-soaked sponge on the surface element, as shown in Figure 5b. Two corrosion rate measurements were taken at each point, and the arithmetic mean was used as the representative value.

## 3. Methodology

### 3.1. Calculation of the Carbonation Coefficient (K_CO_2__)

We have the data to obtain the carbonation coefficient K_CO_2__ for each concrete element since we know its carbonation depth, x (mm), and the age of the building, t (years), at which the measurement was taken. Therefore, we will have the actual carbonation coefficient, K_CO_2__ real = x/$\sqrt{t}$, as discussed in Section 1.2.2.

### 3.2. Calculation of the Initiation Time (t_init_)

According to the previous expression, K_CO_2__ = x/$\sqrt{t}$, if we consider that the carbonation depth, x, has reached the concrete cover, c, of the studied reinforced concrete element, i.e., x = c, we can calculate the initiation time of corrosion (when the aggressor reaches the reinforcement) as t_init_ = (c/K_CO_2__)^2^, as explained in Section 1.2.1.

### 3.3. Calculation of the Propagation Time until Cracking (t_crack,corr_) and the Corrosion Rate (V_corr_) during This Period

According to Section 1.2.2, the propagation time of corrosion until cracking is as follows:

$$t_{\mathrm{crack},\mathrm{corr}}\;=\frac{80.c}{\Phi .Vcorr}$$

In all studied elements, the presence or absence of cracking was analysed. By knowing the total age of the building, t, and the estimated initiation time of corrosion, t_init_, calculated according to Section 3.2, the propagation period of corrosion until cracking can be calculated as t_crack,corr_ = t − t_init._. With knowledge of the concrete cover, c, and the diameter of the reinforcement, Φ, we can determine the corrosion rate by solving the equation studied; therefore, Vcorr = $\frac{80.c}{\Phi .tcrack,corr}$

This way, we will be able to compare the actual corrosion rates based on the specific data from each building with the corrosion rates provided by normative models.

### 3.4. Calculation of the Propagation Time until an Unacceptable Loss of Cross-Section (t_sect,corr_) and the Corrosion Rate (V_corr_) during This Period

In these cases, the reinforced concrete element has experienced a loss of reinforcement cross-section and increased cracking. Similar to the previous case, we can calculate the propagation time of corrosion until the moment of an unacceptable loss of cross-section as t_sect,corr_ *=* t − t_init._. According to Section 1.2.2, using the equation t_sect,corr_ = $\frac{\Delta \Phi }{Vcorr}$, where t_sect,corr_ and ΔΦ are known, the corrosion rate until an unacceptable loss of reinforcement cross-section can be calculated as Vcorr = $\frac{\Delta \Phi }{t_{secc,corr}}$.

In this section, the key determinant is the response to the question: What level of section loss is deemed unacceptable? As mentioned earlier, this decision will depend on the consideration of the ultimate limit state of the structure, involving concepts related to calculation assumptions and the response of structural elements based on their load-carrying capacity. This capacity depends on the safety coefficients employed in the calculations, as well as the allowable stress of the involved materials, such as concrete and steel. Other properties that play a significant role in the overall behaviour of the element include the bond between steel and concrete, and the ductility of the steel. Generally, it is the responsible technical professional who will define the unacceptable level of section loss by evaluating the determining factors specific to each structure. In this research, real scenarios of the propagation period with section losses exceeding 8% have been identified. However, the decision to consider section loss as unacceptable should be made at the discretion of the responsible technician, taking into account the impact of reduced load-carrying capacity and the lack of bond caused by corrosion by-products.

This will allow us to compare the corrosion rate calculated from measurements and real results obtained in situ (as described in the final point of Section 2.4) with the rates predicted by the standard. These calculations can only be performed on elements that have experienced section loss and have reached their ultimate limit state. After evaluating and verifying various structural calculation hypotheses, the type of intervention can be determined: maintenance, prevention, improvement, or strengthening (Supplementary Materials).

**Figure 6:**
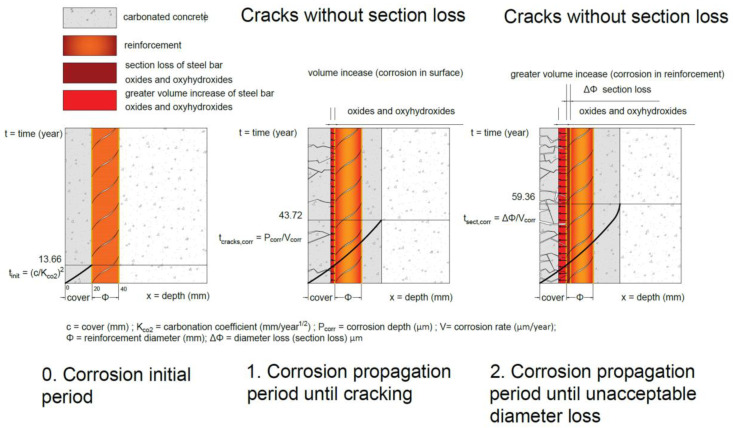
Graphic representation on a concrete section of the different corrosion periods: corrosion initiation (0); propagation until cracking (1); and propagation until an unacceptable loss of diameter (2).

Figure 6 shows a diagram of the corrosion periods in a carbonated reinforcement concrete: initiation period, corrosion until cracking, and corrosion until the loss of diameter which brings the structure to its ultimate limit state (when a section loss is unacceptable).

## 4. Results

### 4.1. Analysis of K_CO_2__

**Scheme 1:**
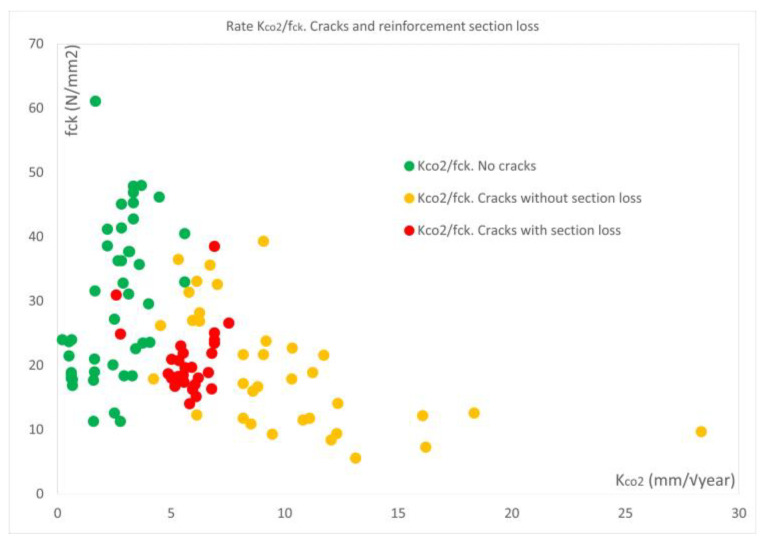
Carbonation coefficient K_CO_2__ and characteristic compressive strength f_ck_.

The different scenarios considered study reinforced concrete elements without cracking (0), elements with cracking but without a loss of reinforcement cross-section (1), and elements with a significant loss of reinforcement cross-section (2). Scheme 1 presents the results of the carbonation coefficient (K_CO_2__) and the characteristic strength (f_ck_) of the 143 elements in the 25 analysed buildings. All the obtained data are provided in an Excel table in the Supplementary Materials.

It can be observed that the lower carbonation coefficients correspond to buildings that have not experienced any pathologies (green dots in Scheme 1), while a higher carbonation of concrete is associated with cracked elements (orange dots). The loss of reinforcement cross-section is seen in reinforced concrete elements with intermediate K_CO_2__ values (around 5 μm/√year, represented by red dots), which are typically found in older buildings, where the carbonation front has reached the reinforcement and corrosion is in full development producing significant section losses; however, K_CO_2__ is inversely proportional to the $\sqrt{t}$ (age of the building), and the aggressive agent (CO_2_) has more difficulty to penetrate into the deeper layers of the concrete (x), so the K_CO_2__ coefficient tends to be more homogeneous in older buildings.

**Scheme 2:**
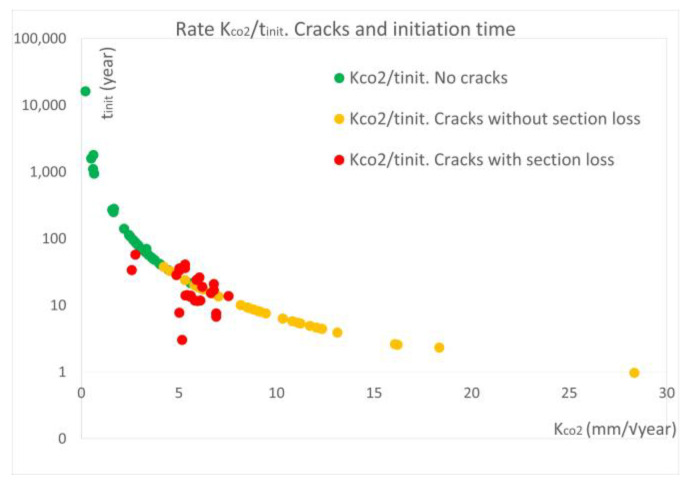
Carbonation coefficient K_CO_2__ and corrosion initiation period t_init_.

**Scheme 3:**
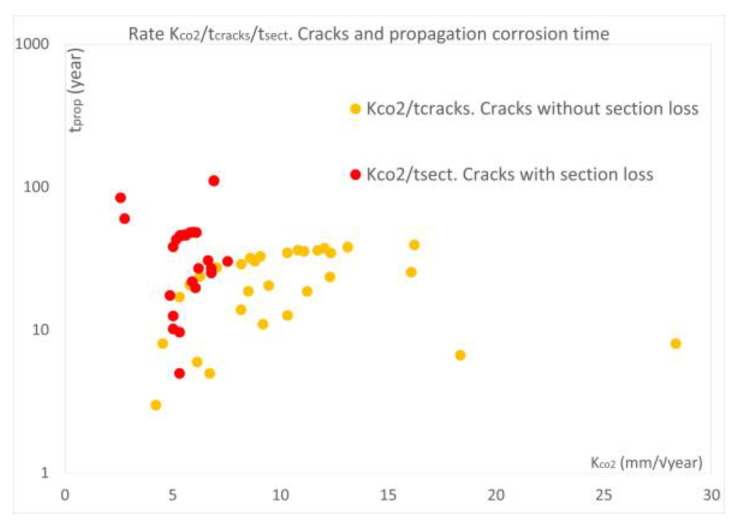
Carbonation coefficient K_CO_2__ and propagation period of corrosion until the cracking of the cover t_tcrack,corr_, and the period until an unacceptable loss of cross-section t_sect,corr_.

In Scheme 2 and Scheme 3, the relationship between K_CO_2__ and the initiation and propagation periods, respectively, is represented for each of the three situations (in the propagation periods, case 0 is not represented as the element does not exhibit corrosion-related pathologies and corrosion has not initiated). In the initiation period, it can be observed that the non-cracked elements and those without a loss of reinforcement exhibit a representation that resembles a quadratic function (green and orange points in Scheme 2; the values are derived from normative formulas relating carbonation depth and time with logarithmic representation), while in the case of the elements with a loss of reinforcement diameter (red points in Scheme 2), it was found that with different coverings to those defined in the project and with a lower covering, the carbonation reaches the reinforcement earlier and the initiation period decreases, and vice versa. Regarding the propagation times, Scheme 3 shows a higher carbonation rate in early-aged elements (orange dots), where the carbonation processes in the outer zone (covering) are more accelerated because the CO_2_ is more accessible near the concrete surface, and slower in the inner layers, with more difficult access, but the bars have already been surpassed (red dots with very advanced corrosion). In this case, the dispersion is produced by the different corrosion rates in the different environments once the aggressive agent has reached the reinforcement.

### 4.2. Analysis of Corrosion Rate V_corr_

**Scheme 4:**
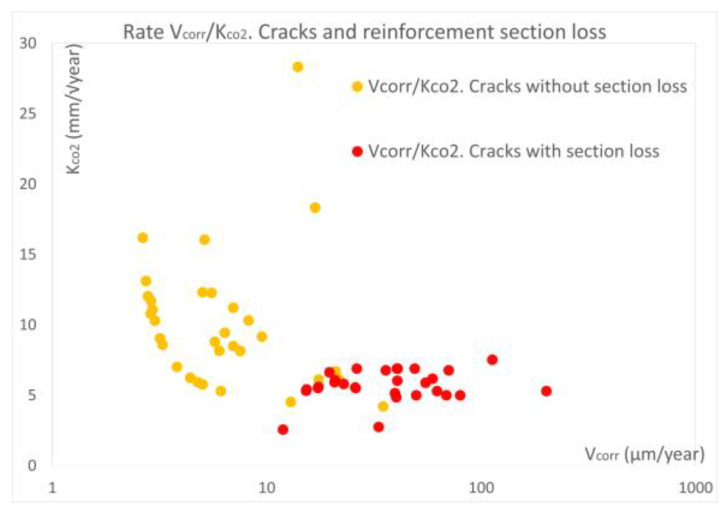
Relationship between corrosion rate V_corr_ and carbonation coefficient K_CO_2__ during propagation periods.

**Scheme 5:**
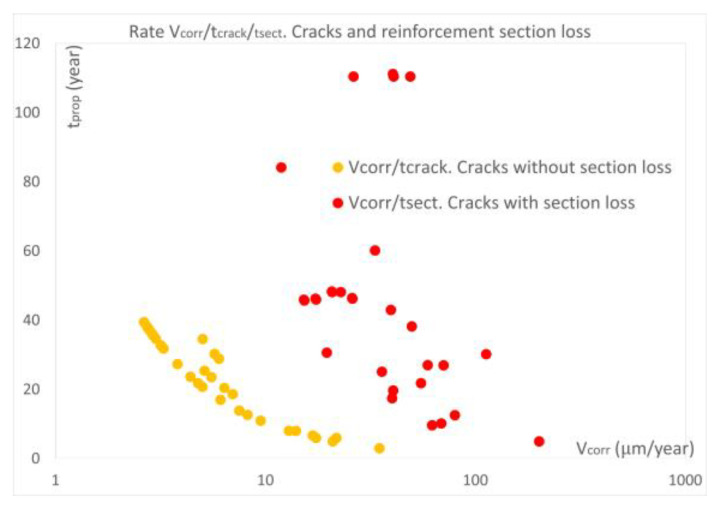
Corrosion rate V_corr_ during the propagation period until cracking of the cover t_crack,corr_ and until the unacceptable loss of cross-section t_sect,corr._

In Scheme 4, a clear trend of lower corrosion rates can be observed in elements that have not experienced any loss of reinforcement (yellow dots), and the carbonation coefficients tend to be higher, following the same trend as discussed in previous figures. Scheme 5 analyses the propagation times until cracking and until loss of section, and the same interpretation can be made. When corrosion is in a “very high” stage of development, it usually coincides with significant propagation times (red dots); the scatter occurs, when there is a loss of section, in V_corr_/t_sect.cracks_ points, because other factors may appear with different typologies and dimensions of the cracks (easy access of H_2_O, CO_2_, temperature, etc.).

### 4.3. Analysis of Average Corrosion Rate V_corr_

In order to systematically analyse all data and compare corrosion rates in each of the considered periods, taking into account all the studied variables (f_ck_, K_CO_2__, age, V_corr_, t_init_, t_prop_) for each of the cases (0, 1, and 2), a statistical analysis has been conducted, identifying the population number (N), the median (M), the arithmetic mean ($\bar{X}$), and the standard deviation (σ), as presented in Table 1.

**Scheme 6:**
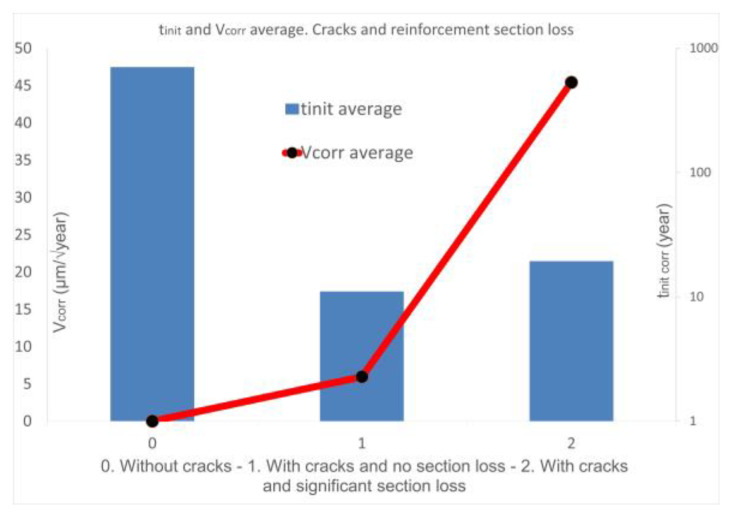
Relationship between corrosion rate V_corr_ and initiation period (0) t_init_.

**Scheme 7:**
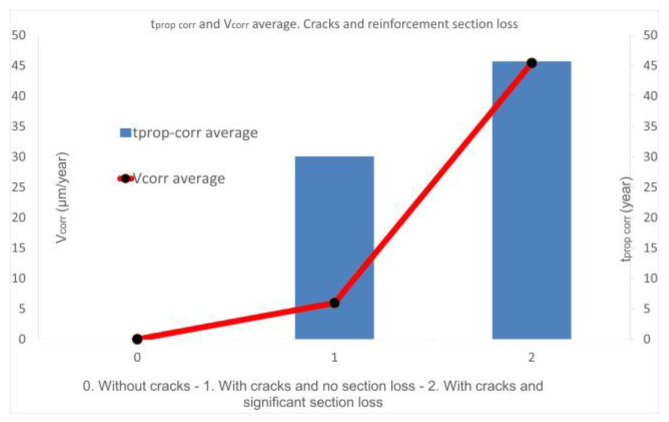
Relationship between corrosion rate V_corr_ and propagation period until cracking (1) t_crack,corr_ and until unacceptable loss of cross-section (2) t_sect,corr_.

The obtained results when comparing the average corrosion rate of the three considered stages and the corrosion initiation time (Scheme 6) show that in case 0 (protected elements, without cracks), elevated initiation times are predicted ($\bar{X}$ = 706.19 years), whereas shorter initiation times are predicted when cracking pathologies occur in case 1 ($\bar{X}$ = 11.06 years) and section loss in case 2 ($\bar{X}$ = 19.41 years); therefore, considering all the elements that have reached corrosion propagation, they had an average initiation time of 13.66 years. With the same trend as in previous studies the corrosion rate in the propagation period up to cracking is “high” ($\bar{X}$ = 30.06 years), and “very high” up to an unacceptable reinforcement loss ($\bar{X}$ = 45.70 years). According to Scheme 7, the propagation time to cracking (case 1) is shorter than the propagation time to unacceptable reinforcement loss (case 2), and the corrosion rate also increases from case 1 to case 2.

**Scheme 8:**
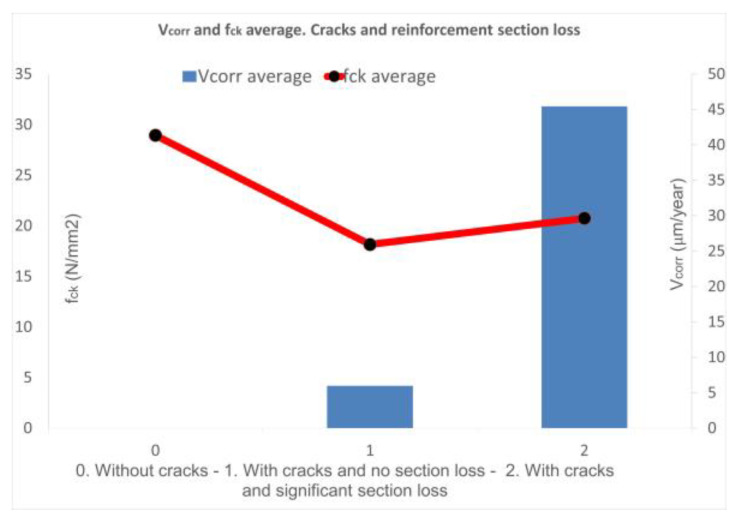
Relationship between corrosion rate V_corr_ and characteristic compressive strength f_ck_.

**Scheme 9:**
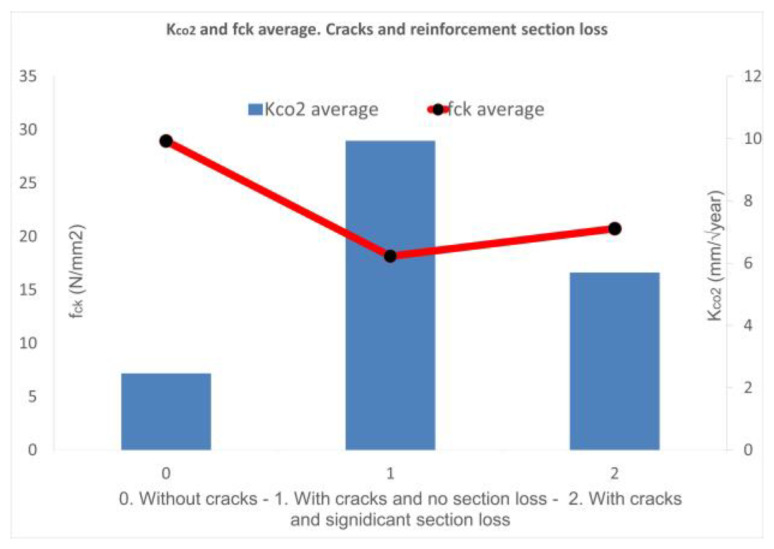
Relationship between carbonation coefficient K_CO_2__ and characteristic compressive strength f_ck_.

In Scheme 8, it can be observed that the trend of zero corrosion rates (indicating the initiation period where corrosion has not started) corresponds to high characteristic strengths (f_ck_). The behaviour during corrosion propagation shows less dependence on f_ck_, but it is evident that there are average corrosion rates of zero in period 0, 5.97 μm/year in period 1, and 45.43 μm/year in period 2. In Scheme 9, it is observed that there is a relationship in all analysed elements where a lower carbonation is associated with higher characteristic compressive strengths (f_ck_) in period 0 (where corrosion has not started). In periods 1 and 2, during corrosion propagation, there is a higher carbonation with a behaviour that is more independent of f_ck_.

### 4.4. Measurement of Corrosion Rate V_corr_

**Scheme 10:**
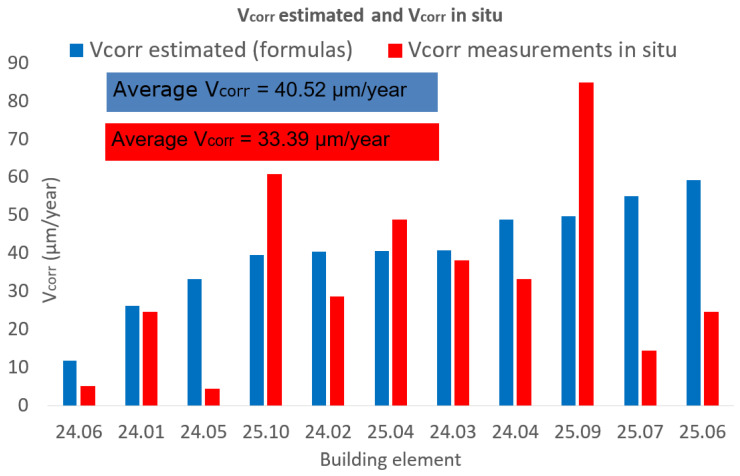
Comparison of estimated corrosion rates V_corr_ according to formulas and measured in situ.

The corrosion rates of all the studied elements have been calculated according to the standard formulas in Section 3.3 and Section 3.4. However, corrosion rate measurements were also performed on 11 elements corresponding to buildings 24 and 25, which had experienced significant corrosion with substantial section loss, in order to compare the measurements obtained from the formulas with the in situ measurements. These data are presented in Scheme 10, and it can be observed that the magnitude is similar between both measurements (ranging from half to double), except for element 24.05. The corrosion rate measured from the diameter loss for element 24.05 was 33.23 μm/year, while the corrosion rate measured in situ was 4.33 μm/year, approximately 8 times lower. Considering the 11 measurements presented in Table 2, the arithmetic average of the corrosion rates calculated using the database obtained and presented in the Supplementary Material with the formulas (Standard Codes) [11,12] was 40.52 μm/year, whereas the in situ measurements with a portable corrosion tester yielded an average corrosion rate of 33.39 μm/year. This confirms that the method of calculations and verified in situ measurements has been appropriate.

As a consequence of the known results in these 25 buildings, recently published studies were carried out with more specific populations, for example, about the position in the building elements [57] or reinforced concrete elements under sanitary slabs [58]. Other studies are currently being carried out in elements exposed outside the buildings, with the aim of improving standard models in each of the possible exposure environments.

## 5. Discussion, Actions, and Intervention

**Scheme 11:**
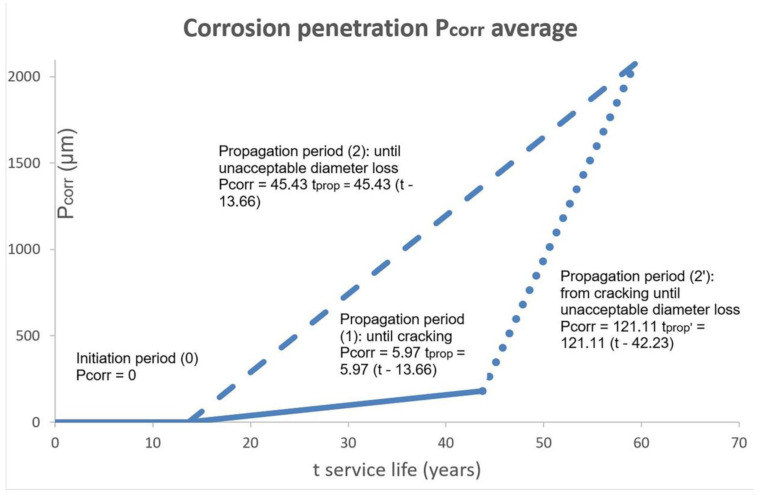
Diagram of section loss (P_corr_) vs. time (t) in different periods and service states.

The analysis of the results provides a clear distinction in the corrosion progression among the three studied scenarios, which can be represented by Scheme 11 with different lines. The slope of each line corresponds to the average corrosion rates of all the studied elements: the first line represents period 0 with a zero slope, the second line represents period 1 with a slope of 5.97, and the dashed line represents period 2 with a slope of 45.43.

The corrosion penetration function in period 1 is P_corr1_ = 5.97 t_prop_ = 5.97 t_crack,corr_ = 5.97 (t − t_init_) = 5.97 (t − 13.66), which corresponds to the propagation period until cracking. The corrosion penetration function in period 2 is P_corr_ = 45.43 t_prop_ − 45.43 t_sect,corr_ − 45.43 (t − t_init_) = 45.43 (t − 13.66), which corresponds to the propagation period until unacceptable diameter loss.

There is an important difference between the slopes (V_corr_) of the lines in periods 1 and 2. If we consider that V_corr_ in period 1 is constant, then we can read that period 2 of propagation until an unacceptable section loss would be the sum of period 1 and another differentiated period in the propagation of corrosion, subsequent to cracking, and that reaches an unacceptable loss of section, represented by the line whose function is P_corr_ = 121.11 t_prop’_ = 121.11 (t − t_init_ − t_crack,corr_) = 121.11 (t − 42.23).

A discussion is initiated to aid in the identification and decision-making process regarding structures affected by corrosion pathology due to carbonation, by connecting the periods of this process with structural limit state and service conditions (Section 1.3). A work field is opened to standardize intervention protocols with possible maintenance and improvement actions for the affected structures, as explained in Supplementary Materials.

## 6. Conclusions

In this study, the elements with carbonation-induced corrosion of the reinforcement had an average corrosion initiation period of 13.66 years, and corrosion propagation to cracking occurred on average 30.06 years later, close to the minimum service age required by the standards (50 years). However, the elements with corrosion up to an unacceptable loss of diameter occurred 45.70 years after initiation, which shows that once the concrete has cracked, the corrosion accelerates in shorter periods. For the same elements, it was also found that the carbonation coefficient K_CO_2__ in the period of propagation until cracking ($\bar{X}$ = 9.93 mm/√year) was higher than that resulting in the period with loss of reinforcement section ($\bar{X}$ = 5.70 mm/√year), when cracking facilitates the arrival of external agents to the reinforcement, although carbonation is hindered towards more internal layers of the concrete. This work shows the difficulty in assigning a model that predicts corrosion rates of reinforcement bars in reinforced concrete due to carbonation. It has been observed that the obtained corrosion rates are higher than those predicted by normative models with variations in the process. For periods of corrosion propagation until concrete cracking occurs, the arithmetic mean of corrosion rates was found to be 5.97 μm/year, which is within the range specified by the codes (between 5 and 1 μm/year). However, for periods of corrosion propagation with significant losses in the reinforcement section, the corrosion rates obtained from the decrease in steel diameter were significantly higher than the values assigned by the Structural Code and Eurocodes for carbonation-induced corrosion. It is evident that the service life of the structure is progressively affected: V_corr_ due to carbonation does not grow linearly, as suggested by the Tuutti model, but rather increases with the propagation of corrosion, which raises the possibility of considering two different stages in the corrosion propagation period: the time elapsed from the initiation of corrosion until the cracking of the element (which coincides with period 1 of this work and the standard) and the time elapsed from the cracking of the element to an unacceptable diameter loss (which modifies period 2 of this work and the standard). This work encourages further investigation into the causes that contribute to variations in corrosion rates of elements exposed to carbonation and experiencing increased cracking during the propagation period, which facilitates the ingress of aggressive agents and leads to a progressive increase in corrosion. Improving the modelling of different corrosion periods and predictions related to the service states of the structure opens up an important field of research that can assist architects and engineers in making informed decisions, with intervention protocols tailored to the specific conditions of the structure.

## Figures and Tables

**Table 1:** Statistical analysis of all variables studied (f_ck_, K_CO_2__, age, V_orr_, t_init_, and t_prop_) in the 143 elements of the 25 buildings and in the three considered periods (0 corrosion initiation, 1 corrosion propagation until cracking, and 2 corrosion propagation until an unacceptable loss of diameter).

Statistical Indicators				
Variable	N	M	$\bar{X}$	σ
f_ck_ (N/mm^2^)				
0	50	24.00	28.94	11.64
1	64	16.25	18.15	9.05
2	29	18.90	20.73	5.07
K_CO_2__($\mathrm{mm}/\sqrt{year}$)				
0	50	2.76	2.46	1.36
1	64	9.06	9.93	4.42
2	29	5.60	5.70	1.11
Age (year)				
0	50	38.00	29.58	17.24
1	64	41.00	41.13	14.40
2	29	60.00	65.10	28.22
V_corr_ (µm/year)				
0	50	0	0	0
1	64	3.86	5.97	5.98
2	29	39.57	45.43	37.87
t_init_ (year)				
0	50	88.91	706.19	2420.59
1	64	8.24	11.06	9.33
2	29	14.07	19.41	12.61
t_prop_ (year)				
0	50	0.00	13.66	0.00
1	64	29.57	30.06	15.46
2	29	45.71	45.70	31.39

**Table 2:** Measurements of V_corr_ using the reinforcement diameter loss (formulas) and in situ measurements taken with a portable corrosion tester.

Building element (number)	24.06	24.01	24.05	25.10	24.02	25.04	24.03	24.04	25.09	25.07	25.06	M median	$\bar{X}$ average
V_corr_-estimated (μm/year)	11.89	26.26	33.23	39.57	40.47	40.60	40.75	48.90	49.74	54.99	59.28	40.60	40.52
V_corr_ (in situ) (μm/year)	5.03	24.57	4.33	60.84	28.67	48.79	38.03	33.11	84.94	14.39	24.57	28.67	33.39

## Data Availability

Data are contained within the article and supplementary materials.

## Supplementary Materials

The following supporting information can be downloaded at: https://www.mdpi.com/article/10.3390/ma17010101/s1.
