# Supplementary material for: Variation of Corrosion Rate, Vcorr, during the Carbonation-Induced Corrosion Propagation Period in Reinforced Concrete Elements
Source: Materials (Basel). 2023 Dec 24;17(1):101. doi: 10.3390/ma17010101 (PMC10779882; doi:10.3390/ma17010101)
Supplement: Supplementary file 1 [file materials-17-00101-s001.zip › materials-2723444-SUPPLEMENTARY_MANUSCRIPT_20231223.pdf]

Supplementary Material

1. Carbonation-induced corrosion

Codes regulating reinforced concrete structures define durability as the nominal service life during which the structure can respond to the physical and chemical conditions of its exposure environment, which can mainly impact the corrosion of reinforcements. The main cause of a generalized attack on a reinforced concrete element is the penetration of CO_2 [13,18,19-32], present in the atmosphere, through the pores of the cover, leading to a decrease in pH to values close to 9 where the passive film is no longer stable.

1.1. Carbonation.

This study is based on the analysis of reinforced concrete elements that were exposed to environmental conditions leading to carbonation-induced corrosion, and the presence of chlorides has not been considered due to their absence from all cases studied. Carbonation reduces the pH of the steel–concrete interface to values close to neutral pH, causing the loss of reinforcement protection and promoting corrosion. The reaction can be expressed as:

$$\mathbf{CO_2 + Ca(OH)_2 \rightarrow CaCO_3 + H_2O} \qquad \text{(S1)}$$

To determine the durability of reinforced concrete concerning the carbonation-induced corrosion of its reinforcements, it is necessary to understand the rate of carbonation penetration, which is measured through the carbonation coefficient K_{co2}.

1.2. The Carbonation Coefficient: Influencing Factors

To calculate the carbonation depth, *x (mm),* we use the mathematical formula $x = K_{co2} \sqrt[n]{t}$, where K_{co2} (mm/year$^{1/n}$) represents the carbonation coefficient that measures the penetration rate of the aggressor under specific environmental conditions and for concrete with specific characteristics [33-36]. The model commonly used in most cases assumes n=2, as applied in the Eurocode [11] and the Structural Code [12], and it is also considered in this research ($x=K_{co2}\cdot\sqrt{t}$).

Several factors [33,34], such as humidity, CO_2 concentration, temperature, concrete quality, water/cement ratio, characteristic strength, porosity, and cement type, influence the carbonation process. Consequently, more compact and stronger concrete offers greater resistance to carbonation.

- Moisture. It is the factor that has the greatest influence on the carbonation rate. Concrete absorbs moisture from the environment. Carbonation is highest at relative humidity (RH) levels of 60-90% and decreases to a minimum outside that range [34,35,37].
- CO_2 concentration. The presence of CO_2 in the air varies from rural to urban areas, but this factor is not highly determinant in the carbonation rate, except in environments with significant concentrations of CO_2 that promote carbonation [12,36,37].
- Temperature. In principle, high temperatures facilitate ion movement, and therefore the possibility of corrosion. However, a decrease in temperature, up to the dew point, can lead to condensation, increasing moisture and thus carbonation. The effect of humidity can have these two distinct aspects.
- Concrete quality. The mixture and proportion of concrete components (aggregate, sand, cement, and water) and their manufacturing process (placing and curing) will influence carbonation based on these factors.

- Porosity. Pores and capillaries facilitate the diffusion of carbon dioxide. The curing process is important as it affects the hydration of the mixture, particularly in the surface layer, which is the one most exposed to water evaporation during curing. This can lead to increased porosity and the inadequate protection of the reinforcement [13].
- Water/cement ratio. In a precast reinforced concrete element, the water/cement ratio (w/c) is not known, as it can only be determined during the concrete production process. However, the w/c ratio has an impact on the porosity of the concrete: a lower w/c ratio results in reduced porosity, which hinders the penetration of carbonation [36]. This proportion directly affects the characteristic strength of the concrete, which increases as the w/c ratio decreases.
- Characteristic strength, f_{ck}. A higher f_{ck} value indicates stronger and denser concrete, which provides better protection against carbonation. In addition, the issue of binder type influence on concrete carbonation jeopardizes f_{ck} as a durability indicator.
- Type of cement. Portland cements, which are more alkaline, can decrease carbonation, while other types of blended cements with additions of fly ash or blast furnace slag have a lower content of $Ca(OH)_2$ in the cement paste. These blended cements have an increased susceptibility to carbonation due to the presence of supplementary cementitious materials that contribute to a lower alkalinity in the paste [28].

1.3. Initiation period of corrosion by carbonation

The period of initiation, according to normative codes, is defined as the time that elapses from the commissioning of the element until the corrosion damage to the reinforcement begins, and the model defined by the standard [11,12] is as follows:

$$\mathbf{t_{init}} = \left(\frac{\mathbf{c}}{\mathbf{Kco2}}\right)^{2} \tag{S2}$$

where c (x=c) is the concrete cover in mm, Kco_2 is the carbonation coefficient in mm/√year, and it measures the penetration rate of carbonation in reinforced concrete.

1.4. Propagation period of corrosion by carbonation

Once the aggressive factor, in this case, CO_2, has carbonated the entire concrete cover of the structural element, the steel loses the chemical protection provided by the alkaline passive layer, leading to a decrease in the pH of the protective film, which loses its stability, initiating the propagation of steel corrosion. This period of corrosion propagation is considered from the onset of corrosion until an unacceptable damage occurs.

The corrosion rate is considered negligible if it is less than 0.1 μA/cm^2; moderate if it ranges from 0.1 to 0.5 μA/cm^2; high if it falls between 0.5 and 1 μA/cm^2; and very high if it exceeds 1 μA/cm^2. These electrochemical measurements can be correlated with gravimetric units through Faraday's Law, which states that

$$\mathbf{m} = \frac{\mathbf{i.t.Pm}}{\mathbf{n.F}} \tag{S3}$$

where

m = mass of corroded steel in grams

i = current intensity in amperes

t = exposure time in seconds

Pm = molecular weight in grams/mol (for iron, 55.85 grams/mol)

n = valence of the element (number of electrons for iron, 2)

F = Faraday's constant (96500 coulombs/mol) (1 coulomb = 1 ampere/second)
For steel, 1 μA/cm² corresponds to a mass loss of approximately 90 g/m².year and a corrosion penetration rate of approximately 11.7 μm/year.
In order to understand the influence of corrosion rate on the reinforcement in a reinforced concrete structure, the following table [38,39] is provided (Table S1).

Corrosion risk	Corrosion rate (*Vcorr*)		
	μA/cm²	μm/year	g/m².year
NEGLIGIBLE	< 0,1	< 1,17	9
MODERATE	0,1 a 0,5	1,17 a 5,85	9 a 45
HIGH	0,5 a 1	5,85 a 11,7	45 a 90
VERY HIGH	> 1	> 11,7	> 90

Table S1. Estimated corrosion rates according to corrosion risk (RILEM).

The Structural Code [12] considers a corrosion rate V_{corr} = 1 μm/year for normal environments with exposure class XC1 (interiors of buildings with low air humidity, RH < 65%; dry or permanently wet environments); V_{corr} = 4 μm/year for exposure class XC2 (elements in permanent contact with water or buried in non-aggressive soil, foundations; humid environments, rarely dry); V_{corr} = 2 μm/year for exposure class XC3 (interiors of buildings with medium or high air humidity, RH > 65%, exteriors protected from rain; environments with moderate humidity); and V_{corr} = 5 μm/year for exposure class XC4 (elements exposed to non-permanent contact with water; environments with cyclic dryness and humidity). These carbonation-induced corrosion rates are categorized as moderate corrosion risk.

1.4.1. Propagation time for cover cracking

The compounds formed because of corrosion (oxides and oxyhydroxides, for instance) occupy a much larger volume than the initial steel, generating lateral thrust stresses on the surrounding concrete, which lead to cracks and fissures parallel to the direction of the steel bars (longitudinal reinforcement and transverse reinforcement). The time elapsed from the onset of corrosion to the cracking of the cover, as defined by the standard, is calculated as follows [11,12]:

$$\mathbf{t_{crack,corr}} = \frac{\mathbf{P_{corr}}}{\mathbf{V_{corr}}} = \frac{\mathbf{80.c}}{\mathbf{\Phi.V_{corr}}} \tag{S4}$$

where
$t_{crack,corr}$ – time from the onset of corrosion to cover cracking, in years;
P_{corr}—limiting corrosion penetration, in μm;
c—thickness of the concrete cover, expressed in mm;
Φ—diameter of the reinforcement, expressed in mm;
V_{corr}—corrosion rate, expressed in μm/year.

1.4.2. Propagation time of corrosion for an unacceptable loss in diameter of the reinforcement

The corrosion processes continue to propagate, causing a reduction in the cross-sectional area of the reinforcement that advances towards the interior of the steel bar, progressively affecting its load-bearing capacity and posing a risk to the structural safety of the element. The time elapsed from the initiation of corrosion until the occurrence of an unacceptable reduction in the cross-sectional area of the reinforcement, as defined by a thickness ΔΦ, can be calculated according to [11, 12]:

$$t_{sect,corr} = \frac{\Delta\Phi}{V_{corr}} \quad \text{(S5)}$$

where

$t_{sect,corr}$—the time elapsed from the onset of corrosion until the occurrence of an unacceptable loss in diameter in the reinforcement, in years;

$\Delta\Phi$—unacceptable variation in diameter of the reinforcement, expressed in μm;

V_{corr}—corrosion rate, expressed in μm/year.

1.4.2.1. Diameter Variation. But what do we consider an unacceptable loss of bar cross-section? In this case, it opens a debate of constructive and structural nature, which is the subject of other parallel investigations being carried out. The reduction in the cross-section of the reinforcement implies a lower mechanical capacity of the reinforced concrete section, which will affect its load-bearing capacity and structural safety. Losses of section below 5% of the diameter can be considered negligible (service limit state); however, from losses more than 10% the structure may be compromised (ultimate limit state). The variation in diameter of the reinforcement ΔΦ during the corrosion propagation period entails a loss of section that provides us with information about the estimation of the state in which the structure is and its load-bearing capacity. The surface area of the nominal section will be $S = \pi\frac{\Phi^2}{4}$; therefore, the loss of section Δ % will be:

$$\Delta\% = \frac{S-S`}{S}.100 = \frac{\left(\pi\frac{\Phi^2}{4}-\pi\frac{\Phi'^2}{4}\right)}{\pi\frac{\Phi^2}{4}}.100 = \frac{\left(\pi\frac{\Phi^2-\Phi'^2}{4}\right)}{\pi\frac{\Phi^2}{4}}.100 = \frac{\Phi^2-\Phi'^2}{\Phi^2}.100 = \left[1-\left(\frac{\Phi'}{\Phi}\right)^2\right].100$$

where Φ′ is the final diameter of the reinforcement that has lost part of its section.

1.5. Structural Limit States

The limit states are defined as situations in which the structure does not fulfil some of the functions for which it has been designed. During the nominal service life of the structure considered in the design, it must be ensured that the calculated value of the structural response is greater than or equal to the calculated value of the effect of actions. If the above condition is not met, we can consider that we are beyond a limit state. Therefore, the corrosion of the reinforcements will influence the conditions of the structural elements and, consequently, their response to the limit states of the structure.

1.5.1. Nominal Service Life

The building and its structure can respond to the normal conditions of use of the building or infrastructure with an adequate response to the effect of actions. We can consider that this period corresponds, in terms of possible reinforcement corrosion, to the initiation period of corrosion.

1.5.2. Serviceability Limit State

The serviceability limit state affects the functionality and use of the structure, the aesthetic aspects of the construction elements, and the comfort of the users. Therefore, in this state, it is necessary to consider the cracking of the reinforced concrete coverings in pillars, beams, and slabs caused by reinforcement corrosion. These pathologies imply repair and maintenance interventions that ensure the serviceability of the structure without compromising its safety. Based on these considerations, the age of the building in its service life until the serviceability limit state is the sum of the initiation period of corrosion and the time for corrosion propagation until cracking of the coverings occurs.

Service Life (Serviceability Limit State) = t_{init}+ $t_{crack,corr}$

1.5.3. Ultimate Limit State

The ultimate limit state affects the stability of the structure and therefore the safety of the building's users. Reinforced concrete structural elements must meet the requirements of safety and structural functionality with appropriate mechanical behaviour under the actions they may be subjected to during their service life. The structure reaches an ultimate limit state when the corrosion of the reinforcement has caused a section loss that is deemed unacceptable to ensure the structural stability (and also leads to a lack of adhesion between the concrete and the steel). In each case, the project will define the ultimate limit state, and thus the considerations regarding what is considered an unacceptable section loss. Therefore, the age of the building in its service life until the ultimate limit state is the sum of the corrosion initiation period and the propagation time of corrosion until the deemed unacceptable section loss occurs.

Service Life (Ultimate Limit State) = t_{init}+ $t_{sect,corr}$

2. Intervention protocols in reinforced concrete structures damaged by corrosion

A discussion is initiated to aid in the identification and decision-making process regarding structures affected by corrosion pathology due to carbonation, by connecting the stages of this process with service conditions and the potential interventions it may require, as represented in Table 2. These interventions can be classified as maintenance, prevention-protection, improvement, and reinforcement-strengthening measures.

2.1. Maintenance actions are aimed at ensuring habitability and functionality conditions, which are related to finishes, installations, and the overall operation of the construction systems. These actions do not have a direct influence on the structure and correspond to the **initiation period**, when the structure is still in its full-service life and functionality.

2.2. Prevention-protection actions aim to protect the structure and extend its service life, either before or after the occurrence of corrosion pathology. These actions can be carried out using corrosion inhibitors, protective coatings for concrete and reinforcement, anti-carbonation paints, stainless steel reinforcement, and the installation of sacrificial anodes. These actions can be implemented during the construction phase, during the initiation period, or during the propagation of corrosion.

2.3. Improvement actions are more closely related to the **periods of corrosion propagation until cracking**, where the structure reaches its service limit state, compromising primarily the requirements of functionality and habitability. In this case, interventions require the repair and/or restoration of the damaged section of the element (such as the application of thixotropic mortar, formwork concrete, etc.). These actions can also be compatible with prevention-protection measures.

2.4. Strengthening actions will be necessary when the structure is compromised and reaches an ultimate limit state that does not guarantee the conditions of stability and safety. We are in the **period of corrosion propagation with a diameter loss of the reinforcement that is not acceptable**. Structural reinforcement projects are required, involving the partial or total replacement of elements

(reinforcement and concrete) or the use of complementary systems (metal structure reinforcement, the jacketing of reinforced concrete structure, carbon fibre reinforcement, etc.).

Corrosion period	Reinforcement - cover	Service state	Conditions	Actions
**Initiation**	**Uncracked**	Nominal service life	Habitability	Maintenance and prevention
**Propagation**	**Cracked**	Serviceability limit state	Security	Improvement and prevention
	**Unacceptable diameter loss**	Ultimate limit state	Ruin	Strengthening and prevention

Table S2. Scheme relating corrosion periods, service states, and intervention on reinforced concrete structures.
